# PET kinetics of radiolabeled antidepressant, [*N*-methyl-^11^C]mirtazapine, in the human brain

**DOI:** 10.1186/2191-219X-1-36

**Published:** 2011-12-15

**Authors:** Ole L Munk, Donald F Smith

**Affiliations:** 1 Department of Nuclear Medicine & PET Centre, Aarhus University Hospital, Nørrebrogade 44, Aarhus C 8000, Denmark; 2 Center for Psychiatric Research, Aarhus University Hospital, Risskov 8240, Denmark

**Keywords:** [^11^C]mirtazapine, antidepressant, PET, kinetic models, distribution volume, binding potential, human brain

## Abstract

**Background:**

We compared six kinetic models with and without the requirement of arterial cannulation for estimating the binding potential of [*N*-methyl-^11^C]mirtazapine in the living human brain.

**Methods:**

Distribution volumes of [*N*-methyl-^11^C]mirtazapine in brain regions were estimated using single- and two-tissue compartment models as well as a graphical plasma input model. The two-tissue compartment model provided a direct estimate of the binding potentials of [*N*-methyl-^11^C]mirtazapine in brain regions, while binding potentials of the single-tissue compartment model and the graphical plasma input model were estimated indirectly from ratios of distribution volumes in brain regions. We obtained also direct estimates of binding potentials using a graphical reference tissue model and two nonlinear reference tissue models.

**Results:**

The two-tissue compartment model required several fits with different initial guesses for avoiding negative values of parameters. Despite the extra fits, estimates of distribution volumes and binding potentials of [*N*-methyl-^11^C]mirtazapine obtained by the two-tissue compartment model were far more variable than those produced by the other methods. The graphical plasma input method and the graphical reference tissue method provided estimates of the binding potential that correlated closely, but differed in magnitude. The single-tissue compartment model provided relatively low estimates of binding potentials with curves that failed to fit the data as well as the three other methods that used the entire series of positron emission tomography data. The reference tissue method and the simplified reference tissue method provided similar, consistent estimates of binding potentials. However, certain assumptions of the simplified reference tissue method may not be fulfilled by the radioligand.

**Conclusion:**

The reference tissue method is appropriate for estimating the binding potential of [*N*-methyl-^11^C]mirtazapine in regions of the human brain so that the binding potential of [*N*-methyl-^11^C]mirtazapine can be estimated without arterial cannulation.

## Background

**Figure 1:**
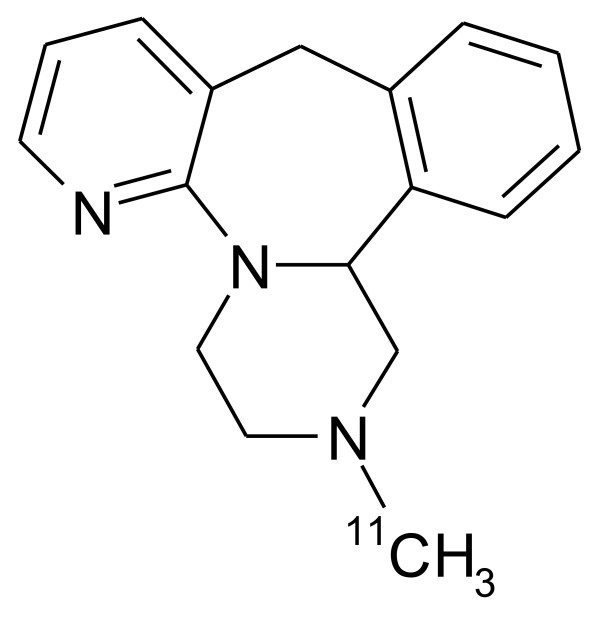
**Structure of [*N*-methyl-^11^C]mirtazapine**.

Mirtazapine is an atypical antidepressant drug belonging to a class of compounds known as noradrenergic and specific serotonergic antidepressants [1-5]. Extensive clinical trials have shown mirtazapine to be among the most effective antidepressants [3,4]. The antidepressant enters the central nervous system rapidly [6], which makes it a suitable candidate for short-term kinetic modeling [7]. Previously, we radiolabeled mirtazapine with ^11^C (Figure 1) and studied it by positron emission tomography [PET] in anesthetized pigs [8,9]. We obtained arterial blood samples for kinetic data analysis and found that the compound had a differential distribution in brain regions, with the highest binding potentials in the frontal and temporal cortices, intermediate binding potential in the thalamus, and low binding potentials in the striatum, hypothalamus, and brainstem. Thereafter, we initiated PET studies with arterial sampling in humans and used a single-tissue compartment model to estimate brain regional binding potentials [10]. We found that regions of the human brain also differed markedly in the distribution and binding of [*N*-methyl-^11^C]mirtazapine, which has furthered our interest in using the radioligand for PET. Experience with arterial cannulation in humans has, however, indicated that the procedure can be disadvantageous for routine brain imaging [7], in part occasionally due to discomfort at the cannulation site. Here, we carried out the present study to determine whether a reference region method, which requires no arterial blood sampling, can also provide reliable estimates of binding potentials of [*N*-methyl-^11^C]mirtazapine in human brain regions.

## Methods

### Subjects

The study was approved by the Danish Medicines Agency, the Ethics Committee of Aarhus Municipality, and the Committee for Good Clinical Practice of Aarhus University Hospital. We used five males (ranges 37 to 66 years old, 70 to 94 kg) who gave informed consent to participate in the study after receiving a written and oral account of the project. They were currently in good general health with no indication of past or present mental illness.

### Scanning procedure

For brain imaging, we used an ECAT EXACT HR PET camera (CTI/Siemens, Knoxville, TN, USA) with a radiation shield located on each side of the neck (NeuroShield^®^, Scanwell Systems, Montreal, Canada). After a transmission scan, subjects received an intravenous injection of [*N*-methyl-^11^C]mirtazapine (ranges: radioactivity injected = 175 to 413 MBq, specific activities = 13 to 67 GBq/μmol, stable mirtazapine dosage = 15 to 50 μg) at the start of a 60-min dynamic PET scan of 28 frames (6 × 10 s, 4 × 30 s, 7 × 60 s, 5 × 120 s, 4 × 300 s, 2 × 600 s) recorded in 3D mode. PET data were reconstructed using filtered backprojection and a Hanning filter with a cutoff frequency of 0.5 per cycles, resulting in a special resolution (FWHM) of about 5 mm. Correction for attenuation was based on a transmission scan. The dynamic PET data were decay-corrected to the scan start.

### Radiochemistry, blood chemistry, and metabolite analysis

[*N*-methyl-^11^C]Mirtazapine was prepared from (±)-*N*-desmethyl mirtazapine (*Z*)-2-butenedioate, and analytical high-performance liquid chromatography [HPLC], determination of radiochemical purity, and product identity were done as described elsewhere [9,11]. Thirty-five blood samples (18 × 10 s, 4 × 30 s, 5 × 1 min, 7 × 5 min, 1 × 15 min) were obtained manually from an antecubital artery and were decay-corrected to the scan start. The fraction of unchanged [*N*-methyl-^11^C]mirtazapine in the plasma was determined with radiodetection by integration of the peak corresponding to the radiopharmaceutical identity and was expressed as a percentage of the total of all radioanalytes recovered by HPLC. Seven radiochemical fractionations of extracts of plasma samples were measured at 1, 2.5, 5, 15, 25, 40, and 60 min. A double-exponential function was fitted to these measurements and was used to estimate the continuous time-course of the radiochemical fractions of [*N*-methyl-^11^C]mirtazapine needed to calculate the metabolite-corrected arterial input function.

### Image analysis

The data of the dynamic [*N*-methyl-^11^C]mirtazapine scan were summed for each subject, and each summed image was coregistered automatically using a software based on the medical image NetCDF [MINC] programming package developed at the Montreal Neurological Institute [MNI]. Briefly, the summed PET scans were converted into the MINC format and were linearly registered to the MNI/International Consortium for Brain Mapping [ICBM] 152 T1 brain template [12]. The transforms were concatenated to produce the transformation used for bringing the dynamic PET images into the MNI/ICBM 152 common standardized space.

Representative regions of interest were obtained automatically from each subject's data by a custom-made software and a segmented atlas of the human brain [13]. Time-activity curves [TACs] were generated from the dynamic PET study for five regions: the cerebellum (region 1), striatum (region 2), hippocampus (region 3), frontal lobe (region 4), and thalamus (region 5).

### Kinetic analyses

Time-activity curves for each subject were analyzed using six kinetic methods: (A) single-tissue compartment model with uncorrected and metabolite-corrected arterial plasma input functions, (B) two-tissue compartment model with uncorrected and metabolite-corrected arterial plasma input functions, (C) graphical plasma input model with metabolite-corrected arterial plasma input function [14], (D) graphical reference tissue model with a cerebellum TAC [15], (E) reference tissue model with a cerebellum TAC [16], and (F) simplified reference tissue model with a cerebellum TAC [17]. Methods A and B use metabolite-corrected arterial plasma curves as input function to the kinetic model, and uncorrected arterial plasma curve including metabolites for the blood volume. The reference tissue models, namely methods D, E, and F, use a cerebellum TAC instead of plasma input functions and do not require blood sampling. Method D can be applied with or without a *k*_2 _correction [15]; we excluded the correction to maintain a linear method without assumptions about the *k*_2 _values.

All models can be described in terms of microparameters: *K*_1 _(ml ml^-1 ^min^-1^) denotes the influx rate constant of the parent compound from the plasma to the free tissue compartment; *k*_2 _(min^-1^) is the rate constant of transfer from the free to the plasma compartment; *k*_3 _(min^-1^) is the rate constant for transfer from the free to the bound compartment; *k*_4 _(min^-1^) is the rate constant for transfer from the bound to the free compartment; and *V*_0 _(ml ml^-1^) is the fractional blood volume in the brain. In methods A and B, we assumed a fixed fractional blood volume of 7% [18]. Estimates of microparameters may be uncertain due to noise. However, data analyses of receptor studies focus on physiologic macroparameters, such as distribution volumes [*V*_T_] (the ratio at equilibrium of the tracer concentration in the tissue to that in the plasma) and binding potentials [BP_ND_] (the ratio at equilibrium of a specifically bound tracer to that of a non-displaceable tracer in the tissue), which are more stable and can be derived in terms of the microparameters. Method A provides estimates of the distribution volumes (*V*_T _= *K*_1_/*k*_2_). In addition, indirect estimates of binding potentials were calculated for the binding regions by relating fitted values for the distribution volume in the binding region to that of the reference region, assuming that distribution volume of the non-displaceable compartment [*V*_ND_] in the receptor-deficient reference region and in the receptor-rich binding region are equal:

(1) $$\text{B}{\text{P}}_{\text{ND}}=\;\left(V_{\text{T}}-V_{\text{ND}}\right)∕V_{\text{ND}}.$$

Method B provides estimates of the distribution volumes (*V*_T _= (*K*_1_/*k*_2_) (1 + *k*_3_/*k*_4_)) and binding potentials (BP_ND _= *k*_3_/*k*_4_). Method C provides estimates of *V*_T _as the slope of a linear regression to the late linear part of the Logan representation. For method C, BP_ND _can be indirectly calculated according to Equation 1. Method D provides estimates of the distribution volume ratio *V*_T_/*V*_ND _as the slope of a linear regression from which the binding potential (BP_ND _= *V*_T_/*V*_ND _- 1) is derived (Equation 1). Methods E and F directly include BP_ND _= *k*_3_/*k*_4 _as a model parameter.

It has been shown for neuroreceptor modeling that weights should not be based on noisy TACs and that uniform weighting is recommended if nothing is known about the noise of the measurements [19]. We tested two simple weighting schemes by comparing kinetic parameters estimated by nonlinear regression with uniform weighting and with weighting by frame duration. Goodness-of-fit was measured by the Akaike criterion [20]. Parameter estimates may fluctuate considerably when fitted by the nonlinear methods A, B, E, and F. We report the best fits and their corresponding parameter estimates that represent the best mathematical representation of the data as found by an automatic optimization routine [21]. For noisy data, the resulting parameters can depend on the initial guess due to local minima, which may be unphysiologic and even include negative microparameters that are not compatible with the kinetic model. In these cases, the data analysis is less straightforward since quality control of the fits is needed. In this study, our only exclusion criterion was the negative parameters, and those fits were remade with a different initial guess. Otherwise, we report parameters from the fits that yielded the lowest Akaike value. Except for the non-negativity constraint, we did not introduce subjective upper or lower limits for parameter estimates. In reality, we only had problems with local minima when using Method B that led to estimates of *k*_3 _and *k*_4 _that were particularly unreliable; its sensitivity to noise was systematically dealt with by making 20 fits using randomized initial guesses and reporting the parameters from the fit with the lowest Akaike value with non-negative parameters. For the other methods, we would get the same physiologically reasonable parameter estimates using any reasonable initial guess. Thus, the extensive procedure using 20 fits was not necessary for the other methods.

We used nonparametric tests (chi-square test, Kruskal-Wallis *H *test, Mann-Whitney *U *test, and Spearman's rho) with Bonferroni correction for multiple comparisons for determining the statistical significance of the results.

## Results

**Figure 2:**
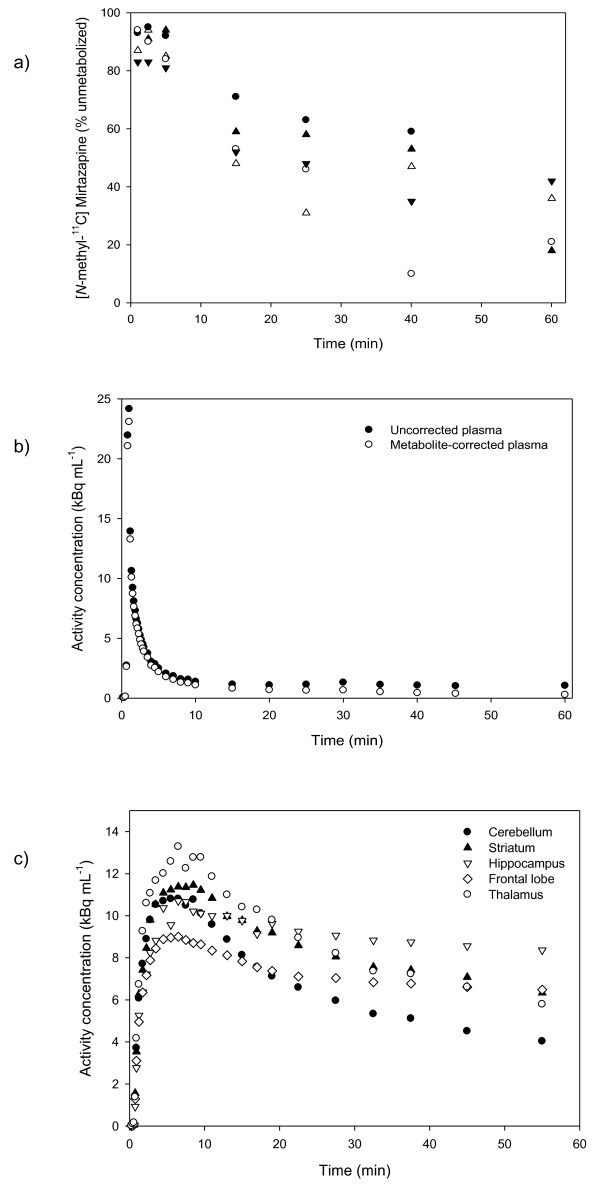
**Time-radioactivity curves for [*N*-methyl-^11^C]mirtazapine in the bloodstream and the brain**. (**A**) Percentage of [^11^C]-derived radioactivity corresponding to the unmetabolized [*N*-methyl-^11^C]mirtazapine in the bloodstream of each subject after intravenous injection. The five symbols correspond to the five subjects. (**B**) Decay-corrected time-radioactivity curves for [^11^C]mirtazapine in the plasma after intravenous bolus injection in one subject. (**C**) Decay-corrected time-activity curves for [^11^C]mirtazapine in brain regions in one subject.

Figure 2 shows time-radioactivity curves for [*N*-methyl-^11^C]mirtazapine in the bloodstream and the brain. Considerable amounts of unmetabolized [*N*-methyl-^11^C]mirtazapine remained in the bloodstream throughout the scan, with 30% to 60% of the radioactivity in the bloodstream arising from unmetabolized [*N*-methyl-^11^C]mirtazapine at a 25-min postinjection and 20% to 40% of [^11^C]-derived radioactivity stemming from an unmetabolized parent compound at the end of the 60-min scan. The range of values of [*N*-methyl-^11^C]mirtazapine in the bloodstream tended to increase with time, perhaps due partly to uncertainties in detecting the compound as radioactivity gradually declined.

Table 1 shows the distribution volume of [*N*-methyl-^11^C]mirtazapine estimated by methods A, B, and C. Statistical analysis of the data indicated that the weighting procedure failed to significantly affect the estimates of distribution volumes (Mann-Whitney *U *two-tailed test, *p *= 0.85), so the data obtained with and without weighting were pooled and used subsequently. Since the two-tissue compartment model sometimes produced negative values for kinetic parameters, additional fits were made in order to always obtain a positive estimate of the distribution volume of [*N*-methyl-^11^C]mirtazapine. Despite that procedure, the statistical analysis confirmed that the estimates of distribution volumes provided by the three methods differed significantly (χ^2 ^= 24.4, df = 2, *p *= 0.001), and the table shows that the two-tissue compartmental model produced higher and more variable values than those provided by methods A and C.

**Table 1:** Distribution volume of [*N*-methyl-^11^C]mirtazapine estimated using three methods

Method	Region	Subject				
		**1**	**2**	**3**	**4**	**5**
A	1	6.3	7.9	5.4	6.6	6.5
	2	9.0	11.2	9.6	10.2	10.2
	3	11.9	14.1	10.7	13.3	11.7
	4	8.6	10.6	10.5	9.8	10.3
	5	8.8	10.8	9.1	10.1	9.4
B	1	297.9	7.9	5.4	8.6	7.2
	2	25.2	11.6	9.7	33.4	18.3
	3	187.1	14.1	10.9	39.1	14.0
	4	26.5	34.3	11.0	31.6	17.3
	5	19.2	10.8	9.2	169.5	39.9
C	1	7.7	7.7	5.2	9.0	6.9
	2	11.9	11.4	9.6	14.2	11.8
	3	17.8	15.0	10.6	25.9	13.4
	4	13.8	11.7	11.4	18.5	13.1
	5	10.8	10.4	8.9	13.7	10.2

Method A is the single-tissue compartment model with uncorrected and metabolite-corrected arterial plasma input functions. Method B is the two-tissue compartment model with uncorrected and metabolite-corrected arterial plasma input functions. Method C is the graphical plasma input model with metabolite-corrected arterial plasma input function. Region 1 is the cerebellum, region 2 is the striatum, region 3 is the hippocampus, region 4 is the frontal lobe, and region 5 is the thalamus.

Table 2 shows estimates of the binding potentials of [*N*-methyl-^11^C]mirtazapine obtained by the six methods. The weighting procedure failed to significantly affect the estimates of binding potentials (Mann-Whitney *U *two-tailed test, *p *= 0.68), so the data obtained with and without weighting were pooled for each method and used for subsequent statistical tests. The statistical analysis confirmed that the estimates of binding potentials of [*N*-methyl-^11^C]mirtazapine provided by the six methods differed significantly (χ^2 ^= 64.4, df = 5, *p *< 0.001), and it is evident from the table that the values obtained by the two-tissue compartment model differed markedly from those provided by the other methods.

**Table 2:** Binding potential of [*N*-methyl-^11^C]mirtazapine estimated using six methods

Method	Region	Subject				
		1	2	3	4	5
A	2	0.44	0.41	0.78	0.54	0.56
	3	0.99	0.78	0.99	1.00	0.80
	4	0.36	0.34	0.94	0.47	0.57
	5	0.40	0.36	0.69	0.51	0.44
B	2	2.94	0.35	34.3	3.58	1.34
	3	26.1	5.71	23.4	4.53	0.98
	4	4.90	3.72	11.3	5.22	1.90
	5	1.74	0.25	10.0	21.6	3.77
C	2	0.55	0.47	0.83	0.58	0.70
	3	1.32	0.94	1.04	1.89	0.93
	4	0.81	0.51	1.17	1.06	0.88
	5	0.41	0.34	0.70	0.53	0.46
D	2	0.42	0.35	0.77	0.48	0.57
	3	0.78	0.61	0.91	0.88	0.72
	4	0.38	0.27	0.98	0.43	0.59
	5	0.38	0.29	0.69	0.45	0.43
E	2	0.52	1.16	0.91	0.61	0.66
	3	1.28	1.53	1.07	1.15	0.98
	4	1.01	2.18	1.79	0.79	1.62
	5	0.38	0.32	0.72	0.45	0.45
F	2	0.51	0.85	0.99	0.56	0.97
	3	1.10	1.06	1.32	1.14	1.05
	4	1.58	2.67	3.11	1.08	2.85
	5	0.39	0.32	0.82	0.47	0.45

Method A is the single-tissue compartment model with uncorrected and metabolite-corrected arterial plasma input functions. Method B is the two-tissue compartment model with uncorrected and metabolite-corrected arterial plasma input functions. Method C is the graphical plasma input model with metabolite-corrected arterial plasma input function. Method D is the graphical reference tissue model with a cerebellum time-activity curve. Method E is the reference tissue model with a cerebellum time-activity curve. Method F is the simplified reference tissue model with a cerebellum time-activity curve. Region 2 is the striatum, region 3 is the hippocampus, region 4 the is frontal lobe, and region 5 is the thalamus.

Table 3 compares the values for the binding potentials of [*N*-methyl-^11^C]mirtazapine obtained by pairs of methods. The statistical analysis showed that the values obtained by the two-tissue compartment model (i.e., method B) were significantly higher than those obtained by each of the other methods. Moreover, the graphical reference tissue model (i.e., method D) produced values of the binding potential that were significantly lower than those obtained by the graphical plasma input model, the reference tissue model, and the simplified reference tissue model (*p *values < 0.05), while the binding potential values obtained by the graphical plasma input method, the reference tissue model, and the simplified reference tissue model did not differ significantly.

**Table 3:** Comparisons of binding potentials of [*N*-methyl-^11^C]mirtazapine estimated by six methods

Method^a^	B	C	D	E	F
A	5.3*	2.5	0.9	3.5*	3.9*
B		4.7*	5.5*	4.1*	3.7*
C			3.2*	1.4	2.0
D				4.0*	4.4*
E					0.6

^a^See the legend of Table 2 for a description of the methods. The nonparametric statistical comparison (*z*-scores) in the table denotes the degree of difference between the binding potential values provided by the methods; *the binding potentials obtained by the two methods differed significantly (two-tailed tests, Bonferroni correction for multiple comparisons, *p *≤ 0.0016).

Table 4 presents correlations between the values for the binding potential of [*N*-methyl-^11^C]mirtazapine obtained by pairs of methods. The values obtained by the single-tissue compartment model (i.e., method A) correlated significantly with those obtained by the graphical plasma input model and the graphical reference tissue model (i.e., methods C and D, respectively). In addition, the values obtained by the graphical plasma input model correlated significantly with those obtained by the graphical reference tissue model and the simplified reference tissue model. A reliable correlation also occurred between the reference tissue model (method E) and the simplified reference tissue model (method F) for the binding potential values.

**Table 4:** Correlations between binding potentials of [*N*-methyl-^11^C]mirtazapine obtained by six methods

Method^a^	B	C	D	E	F
A	0.52	0.83*	0.98*	0.39	0.42
B		0.56	0.60	0.27	0.35
C			0.83*	0.60	0.72*
D				0.38	0.45
E					0.90*

^a^See the legend of Table 2 for a description of the methods. The nonparametric Rho scores in the table denote the correlation between the binding potential values provided by the methods; *statistically significant correlations (two-tailed tests, Bonferroni correction for multiple comparisons, *p *≤ 0.0016).

Table 5 compares Akaike values for fits of the data by methods A, B, E, and F, the methods that use all data points for estimating the binding potential. The weighting procedure failed to affect the Akaike values significantly although there was a tendency for weighing to reduce the Akaike scores (Mann-Whitney *U *two-tailed test *p *= 0.08). The data obtained with and without weighting were pooled for subsequent statistical tests. The Akaike values obtained by methods A, B, E, and F differed significantly (mean ± s.e.m. 77 ± 3, 41 ± 6, 18 ± 5, and 23 ± 5, respectively; Kruskal-Wallis *H *test, *p *< 0.001). Subsequent statistical analysis showed that the Akaike values obtained by method A, the single-tissue compartment model, were significantly greater than those obtained by the other three methods (*p *values < 0.05). On the other hand, the Akaike values provided by the reference tissue model (method E) were significantly smaller than the scores obtained by the two-tissue compartment model (method B) (*p *< 0.05).

**Table 5:** Comparisons of Akaike values for nonlinear fits of [*N*-methyl-^11^C]mirtazapine-PET data

Method^a^	B	E	F
A	4.7*	6.9*	7.1*
B		2.9*	2.5
E			0.5

^a^See the legend of Table 2 for a description of the methods. The nonparametric statistical comparison (*z*-scores) in the table reflects the degree of difference between the binding potential values provided by the methods being compared. *Akaike values obtained by the two methods differed significantly (two-tailed tests, Bonferroni correction for multiple comparisons, *p *≤ 0.0042). A nonsignificant *z*-score indicates that the Akaike values provided by the two methods did not differ reliably.

## Discussion

Central actions of psychotropic drugs continue to be of interest in PET brain imaging [22-24]. Our work shows that mirtazapine, an effective antidepressant drug, has favorable properties for PET brain imaging when the compound is radiolabeled with ^11^C in the *N*-methyl position [10,25,26]. As far as we know, [*N*-methyl-^11^C]mirtazapine is the only radioligand of a popular antidepressant drug that is suitable for PET imaging of the brain in humans. We realize, of course, that mirtazapine affects multiple receptor systems, including alpha-adrenergic, histamine type 1, and serotonin type 2 [5,27,28] receptors. Some may view the lack of receptor specificity of [*N*-methyl-^11^C]mirtazapine as a disadvantage for PET neuroimaging, whereas we view the radioligand as a potential screening device for assessing multireceptor disorders in the living human brain.

Several methods are currently in use for studying the pharmacokinetics of PET radioligands [29,30]. Of particular interest for the present report are methods requiring no arterial cannulation.

We have, therefore, compared kinetic models, with and without the requirement of arterial cannulation, for estimating the distribution volume and binding potential of [*N*-methyl-^11^C]mirtazapine in the living human brain. In a previous study, we used method A, the single-tissue compartment model with arterial cannulation, for assessing the pharmacokinetics of the radiotracer. That method has few parameters and typically provides stable fits with reproducible estimates of parameters. However, some volunteers experienced pain at the site of cannulation. In addition, the relatively high Akaike scores found for that method indicate that the single-tissue compartment model may oversimplify the dynamics of [*N*-methyl-11C]mirtazapine data, perhaps resulting in biased estimates of parameters. The values for the binding potential of [*N*-methyl-^11^C]mirtazapine obtained by method A were, for instance, markedly lower than those obtained by the other nonlinear methods assessed in the present study (i.e., methods B, E, and F). Moreover, the estimates of binding potentials were poorly correlated to those of the other nonlinear methods.

The two-tissue compartment model, method B, provided better fits than method A of the PET data for [*N*-methyl-^11^C]mirtazapine, judging from Akaike scores. The fits of method B were, however, highly sensitive to the initial guess and often had to be redone in order to obtain non-negative estimates of parameters. Furthermore, the microparameters *k*_3 _and *k*_4 _were poorly determined by method B, which lead to variable estimates of *V*_T _and BP_ND _that differed markedly from the values obtained with other methods. This lack of robustness of method B limits its use for modeling of the [*N*-methyl-^11^C]mirtazapine data. It is noteworthy, however, that method B described the data very well, judging from Akaike values, which suggests that at least two-tissue compartments are kinetically distinguishable for [*N*-methyl-^11^C]mirtazapine, namely free and specifically-bound ligand, assuming that the free and nonspecifically bound compartments reach equilibrium rapidly. One could speculate that a slower, nonspecific component of binding might also be present using a third compartment for the radioligand, but we did not examine that model in the present study, in part due to uncertainties that can arise from an excessive number of parameters.

**Figure 3:**
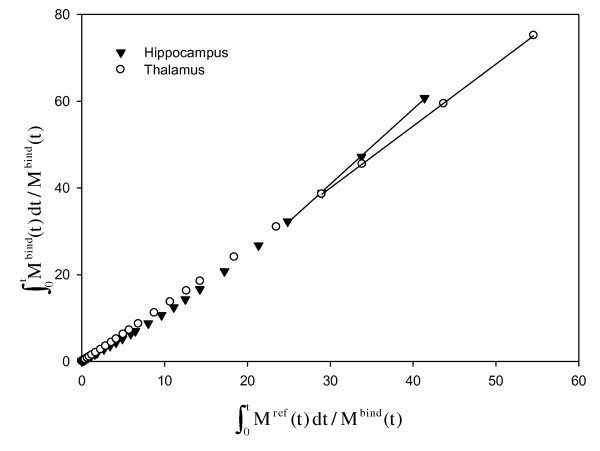
**Decay-corrected time-radioactivity curves for [*N*-methyl-^11^C]mirtazapine in the thalamus and hippocampus fitted by method D**. Method D is the graphical reference tissue model. Note in the Logan representation that the data of the thalamus become linear within 60 min, whereas the data of the hippocampus exhibit a curvature for a longer time.

The graphical linear models, methods C and D, provide estimates of macroparameters that are independent of the underlying compartment scheme. We found that the values of binding potentials provided by methods C and D were reliably correlated. In addition, BP_ND _estimates using method C were similar to those obtained by the nonlinear reference region methods E and F. In contrast, method D provided BP_ND _values that were markedly lower than those obtained with methods C, E, and F, and the estimates of binding potential provided by method D were not reliably correlated to those obtained using methods E and F. Thus, the estimates of BP_ND _provided by method C corresponded better than those of method D to BP_ND _values of [*N*-methyl-^11^C]mirtazapine obtained by the nonlinear methods. However, several factors affect BP_ND _values obtained by the standard implementations of methods C and D used in the present study [14,15,31]. Firstly, exclusion of vascular volume in method C causes distribution volumes to be overestimated and binding potentials to be underestimated, although the bias may be small [14]. In accordance with that, we found in supplementary studies that the effect was of minor importance for [*N*-methyl-^11^C]mirtazapine, being less than 3%. Secondly, exclusion of the *k*_2_-correction term for method D can cause underestimation of the *V*_T _ratio [15], and noisy data can cause slopes to be underestimated by both methods [31]. Thirdly, because methods C and D involve fitting of the slope of the linear part of the Logan plot, curvature of the plot throughout the duration of scanning impairs the estimation of the binding potential (see Figure 3). Since the binding of [*N*-methyl-^11^C]mirtazapine may be relatively slow in some brain regions, methods C and D may have underestimated the distribution volumes and binding potentials in such regions under the conditions of the present study. Perhaps lengthening the duration of the scanning interval could minimize this potential source-of-error so that methods C and D could be used routinely for estimating the binding potential of [*N*-methyl-^11^C]mirtazapine.

The reference tissue model (method E) and the simplified reference tissue model (method F) are nonlinear procedures that rely on the entire data set. The BP_ND _values obtained by the two methods were reliably correlated and did not differ significantly. The present findings show that methods E and F described the data better than methods A and B, judging from Akaike scores. This could be partly due to the variance inherent in the analysis over time of rapidly decaying radionuclides in the bloodstream.

**Figure 4:**
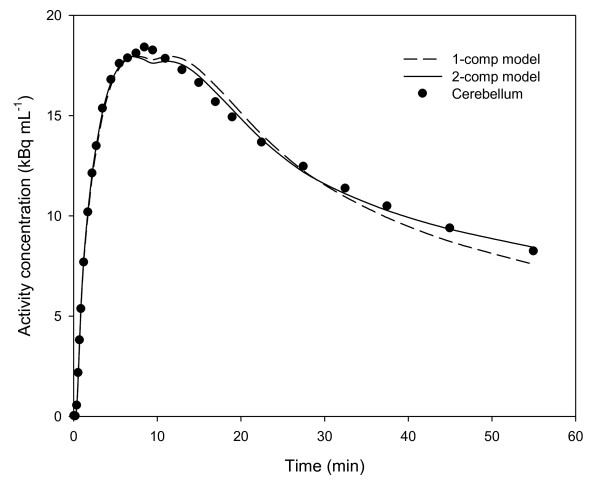
**Decay-corrected time-radioactivity curve for [*N*-methyl-^11^C]mirtazapine in the cerebellum of a single subject**. Note that the data are fitted better by method B (two-tissue compartment model; Akaike score, 31.8) than by method A (single-tissue compartment model; Akaike score, 46.1).

Methods E and F use the cerebellum as a tissue-reference region for the indirect input function of [*N*-methyl-^11^C]mirtazapine, based on previous findings [9]. The present findings show, however, that [*N*-methyl-^11^C]mirtazapine in the cerebellum may be described by two compartments (see Figure 4): a free compartment and a small compartment of nonspecific binding. If two compartments are present for [*N*-methyl-^11^C]mirtazapine in the cerebellum, then methods E and F may underestimate the binding potential of the radioligand [16]. Unlike method E, method F requires that rates of exchange between the free, possibly nonspecific, and specific compartments are so fast that they are kinetically indistinguishable [17]. That assumption may be incorrect for [*N*-methyl-^11^C]mirtazapine because multiple components may have been identified by methods A and B (see Figure 3), making method F less appropriate than method E for estimating regional binding potentials in the living human brain.

In this paper, we have compared estimates of kinetic parameters using [*N*-methyl-^11^C]mirtazapine data in a homogenous group of volunteers. Six models were evaluated based on their robustness and by statistical comparisons of their parameter estimates and ability to describe data. In a future work, a comparison study between different groups of subjects could be used to further validate the parameter estimates and the model selection.

## Conclusions

Taken together, the present findings indicate that the reference tissue model is appropriate for use in PET imaging for obtaining estimates of pharmacokinetic parameters, such as the binding potentials of [*N*-methyl-^11^C]mirtazapine in regions of the living human brain. Since that method does not depend on metabolite-corrected plasma input functions, we conclude that the binding potentials of [*N*-methyl-^11^C]mirtazapine in brain regions can be estimated without arterial cannulation by PET in humans.

A shortcoming of the present study concerns complications that can arise in the kinetic analysis of compounds studied as racemates [32]. However, the enantiomers of [*N*-methyl-^11^C]mirtazapine failed to show marked differences in the binding kinetics in laboratory animals and healthy humans [26,33]. We conclude, therefore, that analysis of PET data using the reference tissue model for racemic [*N*-methyl-^11^C]mirtazapine can provide insight into antidepressant actions that cannot otherwise be studied in the living human brain.

## Competing interests

The authors declare that they have no competing interests.

## Authors' contributions

OLM implemented the kinetic models and performed the kinetic analyses. DFS performed the experiments and statistical analyses. Both authors wrote and approved the final manuscript.
